# A Multi-Faceted Approach of One Teaching Hospital NHS Trust during the *Clostridium difficile* Epidemic—Antibiotic Management and Beyond

**DOI:** 10.3390/antibiotics5010013

**Published:** 2016-02-26

**Authors:** Helena White, Martin Wiselka, David Bell

**Affiliations:** Department of Infection and Tropical Medicine, Leicester Royal Infirmary, Infirmary Square, Leicester LE1 5WW, UK

**Keywords:** *Clostridium difficile* infection, *Clostridium difficile* associated disease, antimicrobial stewardship, care bundle approach

## Abstract

The incidence of *Clostridium difficile* infection (CDI) in the UK rose dramatically during the early years of this century, in part associated with the emergence of the hyper-virulent ribotype 027 strain. The University Hospitals of Leicester (UHL), a 2000-bed acute UK NHS Trust, implemented a number of interventions, which led to an 80% reduction in new cases over a twelve month period. Changes were introduced as a result of collaboration between the Infection Prevention team, the departments of Microbiology and Infectious Diseases, and with the support of the Trust Executive. These strategies are described in detail and included; implementation of antimicrobial stewardship, specific hygiene and cleaning measures, the introduction of a care pathway form for all infected patients, the opening of an isolation ward for patients with CDI, strengthened organisation and clinical management, and rigorous attention to education within the Trust. The implementations described are of continued relevance in the face of new infection challenges, such as the increasing incidence of multi-drug resistant organisms.

## 1. Introduction

*Clostridium difficile* infection (CDI) (syn. *Clostridium difficile* associated disease, CDAD) is one of the most important healthcare-associated infections, in terms of incidence, associated morbidity and mortality, and economic impact [1]. In common with most other UK hospital Trusts, the University Hospitals of Leicester (UHL) experienced a dramatic increase in cases over three years to the end of 2006, reaching a peak of over 170 new cases per month. Over the same period, clinicians in the UK, and elsewhere in Northern Europe and North America, noticed a rise in mortality and other complications associated with the disease [2,3,4]. This rise in mortality was associated with epidemiological data showing the emergence of a previously uncommon but more virulent strain of the organism known as North American pulse field type 1 or ribotype 027 (NAP1/027) [5]. This strain caused increased numbers of cases, and greater severity of disease by virtue of increased toxin production [6], as well as enhanced resistance to commonly used classes of antibiotics [2,7]. The epidemic of hypervirulent CDI in the UK received a great deal of media and public attention [8] and led to the Department of Health requiring mandatory reporting of cases by Trusts for patients aged over two years [9]. 

UHL has approximately 2000 beds, and serves a population of over one million. In light of the unprecedented increase in the number of cases seen within the Trust in 2006, a number of interventions were implemented over a twelve-month period in an attempt to reduce the impact of CDI. These changes were established as a result of a collaborative effort between the Infection Prevention team, the departments of Microbiology and Infectious Diseases, and with the support of the Trust Executive.

The overall aim of the proposed strategies was to reduce the total number of cases of CDI, and to decrease the morbidity and mortality associated with the illness. This paper describes the multi-interventional approach taken by the Trust, which led to a rapid and long-term decline in the number of cases in CDI. These included: alteration in prescribing practice and antibiotic stewardship, specific hygiene and cleanliness measures; the implementation of a care pathway for all patients diagnosed with CDI, the opening of an isolation ward for patients with CDI, strengthened clinical organisation, and rigorous attention to education within the Trust.

## 2. Methods

### 2.1. Prescribing Practice and Antibiotic Stewardship

Policies for the prescribing of antibiotics within the hospitals were amended to effect a quantitative reduction, as well as a qualitative change in antibiotic use. The former was implemented in August 2006, with the introduction of a five-day duration policy for the treatment of most common infections, including respiratory, urinary, and skin/soft tissue infections. The same month, a proton pump inhibitor (PPI) policy was introduced which limited the use of PPI drugs within the hospital and mandated regular review of prescriptions [10]. Qualitative changes in antibiotic use were introduced in May 2007 with a review of approved and recommended antibiotics by the microbiology and pharmacy departments, to achieve a limitation on the use of common classes of broad spectrum agents (e.g., cephalosporins, quinolones, macrolides) where alternatives exist. “Prescription codes” were issued by infectious diseases and microbiology medical staff to clinicians in other areas of the hospital in order to sanction the use of restricted antibiotics. The codes contained the date, initials of the antibiotic, the number of days for which the prescription was sanctioned, the initials of the individual providing the code, a “letter of the week” and a sequential number. This allowed all codes to be verified by the pharmacists providing the antibiotics to the clinical areas. The infectious diseases, haematology and intensive care departments were exempt from the need for prescription codes, and the first dose of antibiotics, such as carbapenems for severe sepsis in penicillin-allergic individuals, or in those known to be infected with multi-drug resistant gram-negative organisms, was permitted without a code in any area, in order to prevent delays in clinical care. Pre-existing practices included the accessibility of antibiotic guidelines for commonly encountered infections on the hospital intranet, with telephone advice available from a microbiologist 24 h a day. The hospital intranet antibiotic pages were significantly updated and extended, with links to prescribing information for common infections encountered in different clinical areas, including those in critical care, maternity, paediatrics and neonates. Separate sections were included on surgical prophylaxis, empirical treatment of sepsis, antibiotic dosing in renal impairment, information about antibiotic assays, and a link to the British National Formulary.

### 2.2. Hygiene and Cleanliness

Increasingly robust policies for hand hygiene, including a staff, patient and visitor initiative entitled “Stop! Clean your hands”, were introduced in early 2007. This initiative included the display of colourful signs throughout the hospital, both in ward and public areas, and as computer screensavers. An audio message was also played around lift areas, asking people to wash their hands before entering and after leaving ward areas. The initiative was also implemented in community settings such as general practice and dental surgeries, and small community hospitals from July 2007 [11]. Regular hand hygiene audits were performed on the wards by the Infection Prevention team, with feedback to staff members. All staff remain expected to follow the Trust guidelines on dress code, which includes being “naked from the elbow down” [12], with the prohibition of white coats, and wrist and hand jewellery.

In addition to the employment of additional housekeeping staff, steam-cleaning technology was introduced in April 2007, in which individual wards in turn were vacated and deep cleaned before being treated with aerosolised hydrogen peroxide [13].

### 2.3. Implementation of a Care Pathway Form

**Figure 1:**
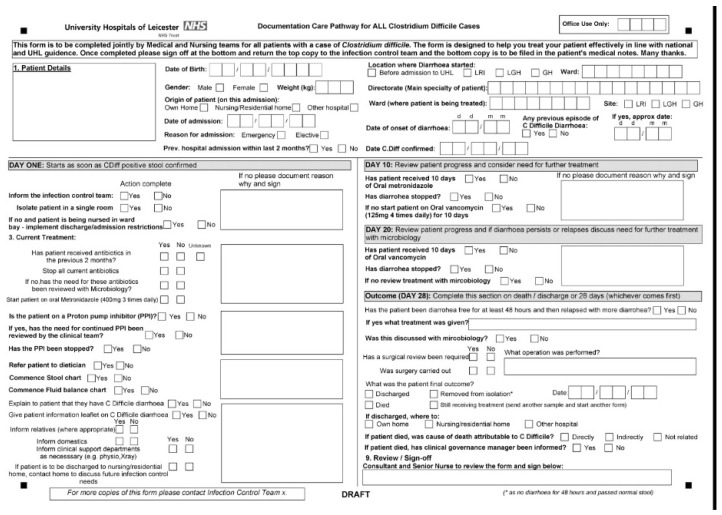
CDI care pathway form.

A paper care pathway was designed in 2007. This was a joint medical and nursing form to be completed for all patients diagnosed with CDI within the Trust (Figure 1). The diagnosis of CDI was made if patients developed new onset diarrhoea and were CD toxin positive, as identified by our hospital laboratory. All positive toxin results were discussed with the treating physicians to determine whether patients had diarrhoea and therefore met the case definition of CDI. If they did, then the care pathway was given to the ward to complete. It acted predominantly as a checklist for clinical staff to ensure that attention was paid to optimising treatment and nutritional support, and assessing the progress and response to treatment at different time points. The aim was to ensure that in whichever directorate the patient was treated, care was standardised. It also acted as a means by which data could be collected from around the Trust. This pathway underwent several revisions over the subsequent two years, incorporating documentation of initial and subsequent treatment regimes for severe or recalcitrant infections. 

### 2.4. Isolation Ward

A reconfiguration of beds within the medical directorate took place on 25 April, 2007, permitting the opening of a dedicated facility for the management of patients with CDI. The facility was a 22-bed combined isolation and cohort ward, with six single rooms, and the remainder arranged in four bedded bays. Patients were cohorted into those with active diarrhoea, and those with recovering disease.

The rationale for the institution of this isolation/cohort ward was several fold; firstly, to reduce transmission of infection within the hospital by physically removing patients with active infection from other patients at high risk; secondly, to develop and improve care for patients with CDI by concentrating clinical expertise in a single area; and thirdly, and of benefit to other areas of the hospital, the formation of the unit facilitated a reduction in pressure on the use of side rooms on the medical wards, use of which had been dominated by the isolation of patients with nosocomial infections.

Patients were transferred to the isolation unit when the diagnosis of CDI was confirmed, and remained on the unit until the time of hospital discharge, or death. Where possible, transfer back to any of the main hospital wards was avoided unless absolutely clinically necessary, such as when expert care was required from another specialty such as surgery.

The unit was staffed with a suitably high nurse to patient ratio (1:4 whole time equivalent), with senior medical support from two consultants in Infectious Diseases and one in Elderly Care. The involvement of allied health staff from dietetics, physiotherapy and occupational therapy enabled concentration on improved nutrition, mobility and rehabilitation prior to discharge. A multidisciplinary team convened weekly, led by the Elderly Care consultant, to facilitate the timely discharge of this often highly dependent group of patients.

### 2.5. Organisation and Clinical Management

The main Trust Infection Control Committee (ICC) met monthly to set Trust policies, and update the Chief Nursing Officer, Medical Director and Chief Executive. During the CDI outbreak other groups were developed, including the Infection Control Operational Group (ICOG), and individual Directorate Infection Control Groups (DICGs). These groups met fortnightly and were responsible for implementing the overarching Trust policies within the different directorates, and auditing compliance. 

In addition, a dedicated Infection Prevention nurse post in CDI was created to help coordinate the Trust’s response to the epidemic. This role involved assessing all patients who met the case definition, ensuring accurate recording of CDI cases, and facilitating the transfer of suitable patients to the isolation ward. The post also carried responsibilities of promoting infection control and treatment guidelines and presenting data at the Trust Infection Control meetings.

### 2.6. Education

The medical directorate employed additional posts to improve education amongst staff, including nursing leads with responsibilities in education and practice development relating to infection control and prevention. The mandatory training programme for clinical staff includes an online or face-to-face module on infection prevention matters, with an additional module on antimicrobial prescribing being compulsory for all medical staff and nurse prescribers. 

## 3. Results

The quantitative antibiotic policy led to an average 20% reduction in defined daily doses (DDD) per 1000-bed days, and the effect of the qualitative antibiotic restrictions policy was to decrease cefuroxime use by 94%, erythromycin by 49% and ciprofloxacin by 38% between May 2007 and April 2008, when compared to the previous twelve months [14]. 

**Figure 2:**
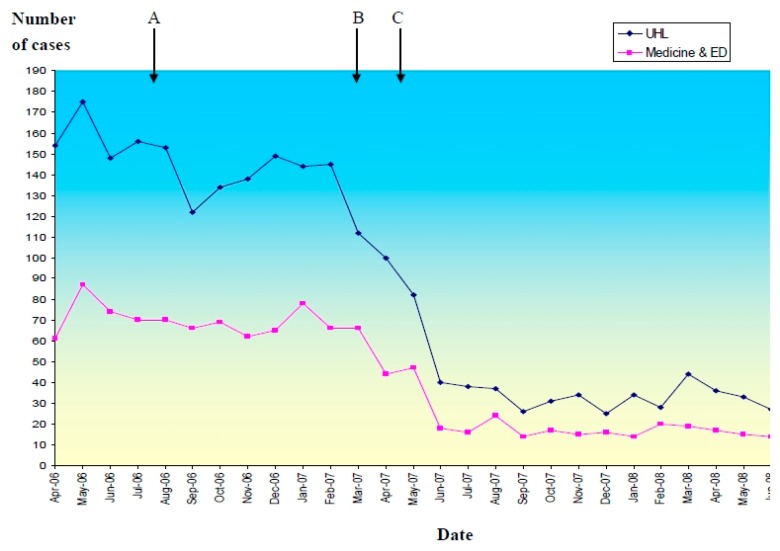
Monthly cases of CDI in the University Hospitals of Leicester (UHL) and timing of intervention. (**A**): quantitative restrictive antibiotic policy and proton pump inhibitor policy introduced August 2006; (**B**): opening of cohort isolation unit, steam cleaning and “stop clean your hands” campaign March–April 2007; (**C**): qualitative restrictive antibiotic policy introduced May 2007.

UHL is a secondary/tertiary level hospital, which is mostly at capacity at all times, and does not have large fluctuations in patient volume. The peak incidence of CDI within the Trust occurred in May 2006 shortly before the introduction of the changes described.A subsequent rapid reduction in the incidence of new cases of CDI was seen, as demonstrated in Figure 2, which shows the total monthly cases for the whole of UHL and within the Medicine and Emergency Department (ED) directorate. From the first quarter of the financial year of 2006–2007 to the corresponding quarter of 2007–2008, there was an 80% reduction in new cases. 

Cases of CDI where patients were discharged from the Medicine and ED directorate were further analysed. Comparisons were made between the patients who were discharged between 25 April 2006 and 24 April 2007, the year before the opening of the isolation/cohort ward, and those who were discharged between 25 April 2007 and 24 April 2008, during the first year of operation of the ward. Data on mortality, length of hospital stay and 28-day emergency readmission rate were obtained and are presented in Table 1.

The data analysis is arranged to allow a comparison between three groups of patients with confirmed CDI:

- Patients diagnosed and managed on medical wards during the year before the opening of the ward (total 787 patients).
- Patients who were managed on the isolation ward for at least part of their inpatient stay, in the first year of its operation (total 216 patients).
- Patients managed on other medical wards during the first year of the isolation ward operation (total 107 patients).

There was no significant statistical difference in the in-hospital mortality rates, median length of hospital stay, or 28-day readmission rate between any of the groups of patients. The in-hospital mortality rates were compared by Fisher’s exact test; A *vs.* B (*p* = 0.07), B *vs.* C (*p* = 0.11) and A *vs.* B + C (*p* = 0.25). The median durations of stay was compared using Mann-Whitney Wilcoxon test; A *vs.* B (*p* = 0.44), B *vs.* C (*p* = 0.44) and A *vs.* B+C (*p* = 0.2). The 28 day emergency readmission rates were also compared by Fisher’s exact test; A *vs.* B (*p* = 0.57), B *vs.* C (*p* = 0.86) and A *vs.* B+C (*p* = 0.49). 

## 4. Discussion and Lessons Learned

This paper describes the multi-interventional approach taken by one UK Teaching NHS Trust in response to the international epidemic of CDI, which peaked in 2006–2007. Details of the implementation of several major interventions have been outlined; including the initiation of an antimicrobial stewardship programme; cohorting infected patients in a single ward area; improved hygiene and environmental cleaning; increased awareness of the infection amongst hospital staff; and strengthened infection control leadership. A PPI policy [10] was also introduced, as PPI use has been associated with increased risk of developing CDI [15,16].

Control of other outbreaks has also been reported following establishment of early case detection methods and implementation of enhanced infection control and other “bundle” measures [17,18]. Since our interventions were implemented in 2006–2007, the UK Department of Health has published core guidance for hospitals, also recommending multi-faceted interventions to prevent and control CDI [3]. Although our interventions were not especially novel, our response to the crisis was prompt and far-ranging, with strategies targeting the clinical management of individual patients, education of staff and the public, and strengthened managerial oversight. Although we were ultimately successful in reducing the cases in our Trust, it seems unlikely that the reduction may be completely explained by any one single intervention and the natural waning of an epidemic must also be considered. No mortality benefit, reduction in length of stay or early readmission rates were seen in those treated on the cohort ward, although the opportunity to concentrate expertise in terms of clinical care may have been beneficial for individual patients, and certainly relieved pressure on the side rooms of other wards. The isolation facility was closed at the end of the epidemic as it was not cost-effective or practical from a staffing perspective to keep this open when the number of cases of CDI had significantly declined. 

The infection control lead nurse post for CDI remains and allows the infrequent cases of CDI seen now across the Trust to be individually reviewed and their care optimised. Antimicrobial stewardship remains an integral part of the management of patients with infections across the Trust, and education for new staff to the Trust continues to include information about the early detection and treatment of those with CDI. The PPI policy also remains in force and is continually revised. Infection control management committees for the different hospital directorates continue to play an important role in the implementation of hospital infection control policies, although the frequency of their meetings was reduced from fortnightly to monthly at the end of the outbreak. The incidence of CDI continues to decline in the Trust; published Public Health England (PHE) data shows the current incidence of CDI in our Trust to be between 2–11 cases/calendar month, with a total of 77 cases diagnosed over the last twelve months [19]. 

We recognise that the interventions possible to control an infection in a large Teaching Trust may exceed those possible in a smaller healthcare facility with fewer resources, particularly the creation of isolation wards, or specialised nursing posts. However, we found that gaining the early support of Executive leadership teams was of paramount importance in our ability to implement effective changes, and agree with others that effective leadership is the cornerstone of success in managing such outbreaks [20,21].

The challenge for our Trust, and indeed for others, will be to direct the same enthusiasm with which we successfully reduced the incidence of the CDI outbreak, towards other healthcare infections, such as the spread of multi-drug resistant gram-negative organisms within our hospitals. Consolidation of infection control procedures, continued support from management steering groups, and heightened awareness amongst medical and nursing staff is vital if these further challenges are to be tackled effectively.

## Figures and Tables

**Table 1:** Outcome measures for patients with CDI managed in different settings in UHL.

	In Hospital Mortality (%)	Median Duration Nights of Stay (Range)	28 Day Emergency Readmission Rate (%)
All Medical wards prior to isolation ward 25/04/2006-24/04/2007	313/787 (39.77)	33 (range 0–445)	104/474 (21.9)
Isolation ward 25/04/2007-24/04/2008	71/216 (32.87)	31 (range 2-242)	35/145 (24.8)
Other Medical wards 25/04/2007-24/04/2008	45/107 (42.05)	29 (range 0–224)	16/62 (25.8)
